# Enhancing the Energy Density of Tricritical Ferroelectrics for Energy Storage Applications

**DOI:** 10.3390/ma12040611

**Published:** 2019-02-18

**Authors:** Li He, Yan Wang, Jinghui Gao, Jianhong Wang, Tongxin Zhao, Zhixin He, Zuting Zhong, Xingmin Zhang, Lisheng Zhong

**Affiliations:** 1 School of Automation and Information Engineering, Xi’an University of Technology, Xi’an 710048, China; heli@xaut.edu.cn (L.H.); 2170320069@stu.xaut.edu.cn (J.W.); 3160434039@stu.xaut.edu.cn (Z.Z.); 2 State Key Laboratory of Electrical Insulation and Power Equipment, Xi’an Jiaotong University, Xi’an 710049, China.; wangyan0616@stu.xjtu.edu.cn (Y.W.); ztx1021@stu.xjtu.edu.cn (T.Z.); hezx24@stu.xjtu.edu.cn (Z.H.); 3 Shanghai Synchrotron Radiation Facility, Shanghai Advanced Research Institute, Chinese Academy of Sciences, Shanghai 201204, China

**Keywords:** ferroelectric material, tricriticality, energy density, breakdown strength

## Abstract

Recently, tricritical ferroelectrics have been drawn tremendous attention, owing to their ultrahigh dielectric permittivities of up to *ε_r_* > 5 × 10^4^, and their consideration for prototype materials in the development of high-performance energy storage devices. Nevertheless, such a materials system suffers from the disadvantage of low breakdown strength, which makes its energy density far from the satisfactory level for practical application. In this paper, a material-modification approach has been reported, for improving the dielectric strength for tricritical ferroelectric materials Ba(Ti_1−*x*_Sn*_x_*)O_3_ (BTS) through doping with Bi_1.5_ZnNb_1.5_O_7_ (BZN) additives. The results suggest that the electric strength has been largely improved in the modified tricritical ferroelectric material (BTS*_x_*-*y*BZN), and the associated energy density reaches *U*_e_ = 1.15 J/cm^3^. Further microstructure investigation indicates that the modified tricritical ferroelectric material exhibits homogenous fine grains with perovskite structure in crystal symmetry, and the BZN may help to form a special structure that could enhance the breakdown strength. The findings may advance the material design and development of high-energy storage materials.

## 1. Introduction

Owing to the ultrafast response of spontaneous polarization with an external electric field, smart material ferroelectrics are considered to be a type of advanced energy storage material, and they can be used for energy harvesting, due to their capacity for rapid storage or release of energy [1,2], and they have possible applications as high-power-density capacitor devices [3,4]. However, such a material system suffers from a disadvantage of low energy density, which limits its utilization as an energy storage device. Therefore, tremendous effort has been made to enhance the energy density for ferroelectric materials [5,6]. 

The energy density that is stored in ferroelectric material can be reflected by the relationship $u_{e}=\int^{\text{​}}EdD=\varepsilon_{0}\int^{\text{​}}Ed\varepsilon_{r}E$, where *E* stands for the electric field strength, and *ε_r_* means the relative dielectric permittivity. These parameters (*E* and *ε_r_*) are two decisive factors for the magnitude of energy density in ferroelectric devices. Recent investigation has reported an ultrahigh dielectric response with a large relative permittivity of $\varepsilon_{r}\approx 2\sim 5.4\times 10^{4}$ in a thermodynamically-special tricritical point, which is tens or thousands of times greater than normal ferroelectric dielectrics, and thus there is a promising future for the potential applications of energy storage devices [7,8,9,10]. Nevertheless, the maximum energy density for tricritical ferroelectrics reported up till now only reaches 30 mJ/cm^3^, which is far below the satisfactory level for practical utilization. It should be noted that main reason for such a drawback is that tricritical ferroelectrics usually exhibit a low breakdown strength, which hinders their application at high electric field strength, making such an ultrahigh-permittivity material system futile for energy storage devices. The central issue to overcoming such a barrier is how to effectively enhance the electric breakdown strength for tricritical ferroelectrics. In the case of dielectric ceramics, tremendous attempts have been made to improve the breakdown phenomenon through various methods. Wang et al., Xie et al. and Zhao et al. studied the role of dopants, such as yttrium, magnesium, gallium, silicon, and silver, in improving the dielectric strength, and thus the energy storage performance of barium titanate-based and correlated materials [11,12,13], and further studies have pointed out the importance of forming a core–shell morphology in the system [14,15]. On the other hand, some work also focused on enhancing the breakdown strength for barium titanate or barium strontium titanate materials, through adding glass into the system [16,17,18]. In addition, intensive studies were performed on forming composite materials with polymers for barium titanate and correlated systems [19,20,21]. Although studies on tricritical ferroelectric and its energy storage properties have been quite limited at present, there is no reason that the above-mentioned approaches on improving energy density should be inapplicable to tricritical ferroelectric materials.

In this paper, we propose a material-modification strategy to enhance the energy density for the tricritical ferroelectric material Ba(Ti_1-*x*_Sn*_x_*)O_3_ (BTS) through doping with Bi_1.5_ZnNb_1.5_O_7_ (BZN) additives [22,23,24]. In order to clarify the effect of BZN on sintering conditions, the density of BTS*x*-*y*BZN has been measured with different sintering temperatures. With the purpose of studying the microstructure and crystal structure of this doped material, scanning electron microscopy (SEM) and synchrotron X-ray diffraction (XRD) have been performed for BTS*_x_*_−_*y*BZN ceramics. Also, the role of tricritical transition on the dielectric response for BZN-doped BaTiO_3_–based ceramics has been investigated, by employing the thermal spectrum of dielectric permittivity for the compositions across the tricritical point. Furthermore, the electric breakdown strength has been evaluated by measuring the polarization (*P*)-electric field (*E*) curves, and comparing the energy storage and energy efficiency of different compositions of BTS*_x_*_−_*y*BZN. The findings may provide a guideline for developing advanced ferroelectrics with large capacities energy storage, and ultrahigh energy efficiency.

## 2. Materials and Methods 

The chemical reagents used are analytical reagent grade (≥99%) produced by Sinopharm Co., Ltd. China. Samples of (1−*y*)Ba(Ti_1-*x*_Sn*_x_*)O_3__−_*y*Bi_1.5_ZnNb_1.5_O_7_ (BTS*_x_*-*y*BZN, *x* = 0.05, 0.105, 0.15, 0.2; *y* = 5 wt %, 10 wt %, 15 wt %, 20 wt %) were all fabricated with the conventional solid reaction method. For Ba(Ti_1__−*x*_Sn*_x_*)O_3_ ceramics, raw powders of BaCO_3_, SnO_2_, and TiO_2_ were first weighed according to the calculated stoichiometric ratio (*x* = 0.05, 0.105, 0.15, 0.2). The mixtures were then ball-milled with anhydrous ethanol for four hours. After being dried in the oven, the samples were calcined in a furnace at 1300 °C for four hours, and then ground into powder. As for the Bi_1.5_ZnNb_1.5_O_7_ ceramics, raw powders of Bi_2_O_3_, ZnO, and Nb_2_O_5_ were weighed, then ball-milled for four hours, and calcined at 800 °C for two hours. With the calculated mixture (*y* = 5 wt %, 10 wt %, 15 wt %, 20 wt %) of prepared BTS and BZN powders, another ball-milling process of eight hours was conducted. Then, 10 wt % polyvinyl alcohol (PVA) was added as a binder. The samples were pressed in a mold, and were obtained after sintering at a series of temperatures of 1025 °C, 1050 °C, 1075 °C, 1100 °C, and 1125 °C for two hours. Finally, two parallel sides of the samples were sputtered with Au electrodes for further measurements.

The crystal structure information was investigated by the synchrotron X-ray diffraction method at beamline 14B1 of the Shanghai Synchrotron Radiation Facility. The specimens were milled into fine powders, and measurements were performed with a photons beam with an energy level of 10 keV. Observations of microstructure and grain size was carried out by scanning electron microscopy (SEM SU3500, HITACHI, Tokyo, Japan). The temperature-dependence of dielectric permittivity was measured by a testing system consisting of a LCR HITESTER (IM3536, Hioki, Nagano, Japan), a Keithley 2000 meter, a Delta chamber, and a set of computers. The LCR HITESTER applies a small alternating current (AC) field with an amplitude of 1 V, at various frequencies on the sample, and it measures the capacity by testing the electrical current. The dielectric permittivity was calculated from the capacity and the dimensions of sample. The Keithley 2000 was used to record the temperature of the sample during the heating or cooling process. Both the LCR and the Keithley were connected to the computer through GPIB interfaces. As for the characterization of energy storage performance, polarization (*P*), electric field (*E*), and hysteresis loop were measured by using a ferroelectric workstation (PK-CPE1701, PolyK, Philipsburg, PA, USA) equipped with a heating specimen stage, and all of the specimens were tested at 40 °C, which is the tricritical point temperature for BTS_0.105_. 

## 3. Results and Discussion

### 3.1. Density

**Figure 1:**
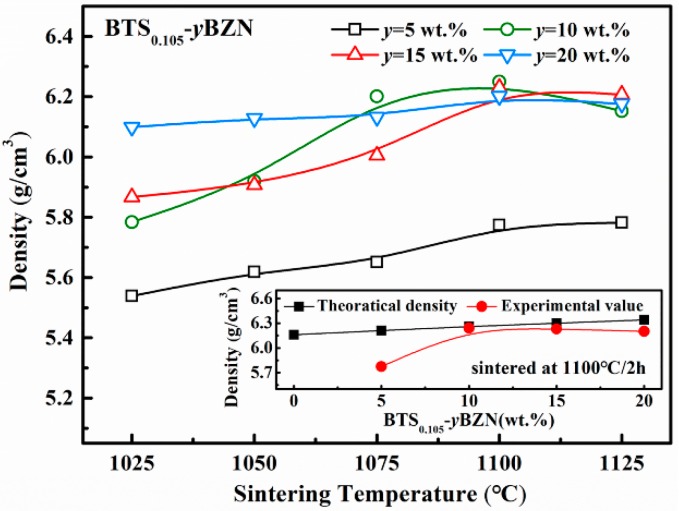
The change of density for BTS_0.105−_*y*BZN ceramics (*y* = 5 wt.%, 10 wt.%, 15 wt.%, 20 wt.%) with respect to the sintering temperature. The inset shows the comparison between the theoretical and experimental densities.

Figure 1 presents the bulk density curves of the BTS_0.105−_*y*BZN ceramics sintered from 1025 °C to 1125 °C. It is obvious that the density change strongly depends on the BZN content, as well as the sintering temperature. It is reported that the sintered BTS_0.105_ ceramic has a bulk density of 6.04 g/cm^3^, with a relative density of 98% at 1380 °C [25], while the BZN ceramic has a lower sintering temperature at 950 °C [26]. Thus, the increase of the BZN doping in the BTS_0.105_ ceramics could effectively lower the sintering temperature. It can be seen in Figure 1 that with 10 wt % BZN added, the optimum sintering temperature of BTS_0.105_-10BZN can be lowered to 1100 °C. The dense and well-sintered BTS_0.105−_*y*BZN(*y* = 10 wt %) ceramics showed a high relative density that was higher than 98%. According to such a result, sintering conditions of 1100 °C, 2 hr were used for the specimen, for further measurements. 

### 3.2. SEM

**Figure 2:**
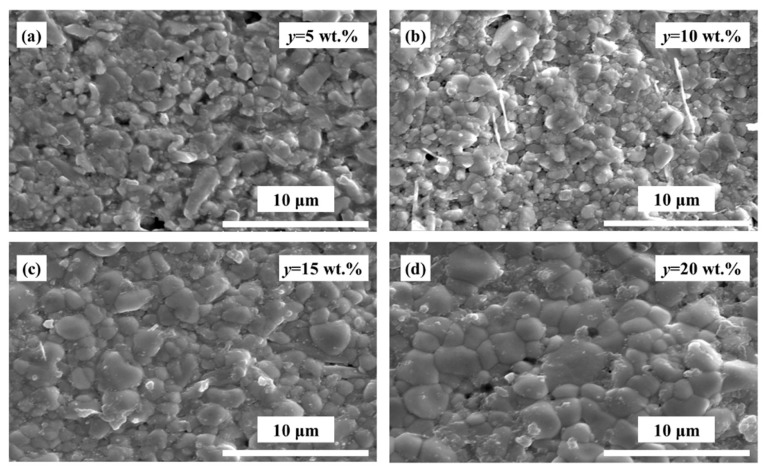
Scanning electron microscopy (SEM) images of the surface morphology for as-grown BTS_0.105_-*y*BZN ceramics (**a**) *y* = 5 wt %; (**b**) *y* = 10 wt %; (**c**) *y* = 15 wt %; (**d**) *y* = 20 wt %.

Figure 2 shows the SEM micrographs of the BTS_0.105_-*y*BZN ceramics with different amounts of BZN, from 5 wt % to 20 wt %. The particle sizes of both BTS_0.105_ and BZN mixing powders after the ball milling process, were about 0.5 μm. According to previous studies, the 950 °C-sintered BZN showed a large grain size of about 10 μm [25,26]. The optimum 1100 °C sintering temperature was higher than that of the BZN (950 °C) [26], but much lower than that of the BZN (1380 °C) [25]. However, no abnormal large BZN grain could be observed in Figure 2. The grain size of each specimen was measured, and the grain size increased from about 0.5 μm to 4 μm, with the BZN content increasing from 5 wt % to 20 wt %. As shown in Figure 2a, large amounts of porosities appeared in the grain boundary, while the dense sintered BTS_0.105_-*y*BZN ceramics (Figure 2b–d) exhibited apparent concrete-like morphologies, with uniform BTS_0.105_ grains surrounded by BZN existing in the grain boundary.

### 3.3. High-Resolution X-Ray Diffraction

**Figure 3:**
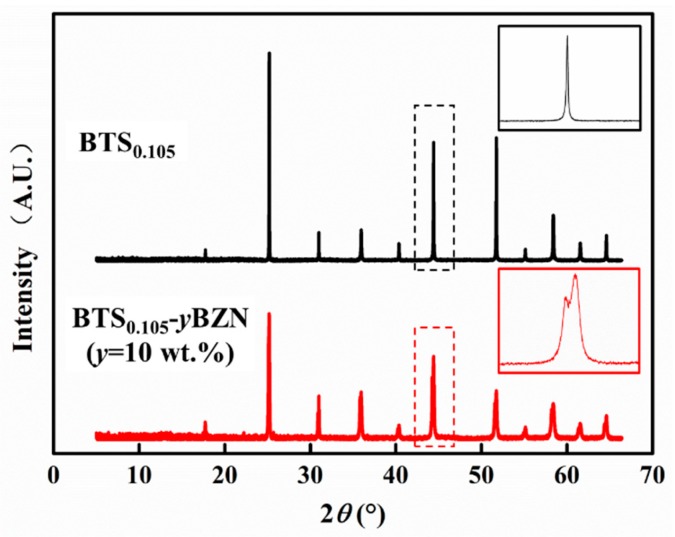
High-resolution X-ray diffraction results for BTS_0.105_ and BTS_0.105−_*y*BZN(*y* = 10 wt %). Each enlarged reflection is shown in the inset.

With the aim of detecting the crystal structure for the BTS_0.105−_*y*BZN system, a high-resolution X-ray diffractometer with a powerful synchrotron light source was employed. The pure BTS_0.105_ with no BZN doping was also scanned as a reference. As shown in Figure 3, the XRD result of BTS_0.105_ suggest that it exhibits a typical perovskite structure for normal ferroelectric material, and the enlarged figure shows a singular peak with no peak splitting. On the other hand, the XRD spectrum for BTS_0.105−_*y*BZN (*y* = 10 wt %) specimen also shows similar peaks with BTS_0.105_, which suggests that BTS_0.105−_*y*BZN (*y* = 10 wt %) exhibits perovskite structure, and there is no obvious BZN phase in the specimen. Moreover, the enlarged reflection shows apparent peak splitting, which means that BZN enters into the lattice of BTS_0.105_ and slightly modifies the dimension of the BTS_0.105_ unit cell. It should be noted that such peak splitting is different from the one in ferroelectric tetragonal, orthorhombic, or rhombohedral symmetry, because it does not vary with different reflections.

### 3.4. Dielectric Response

**Figure 4:**
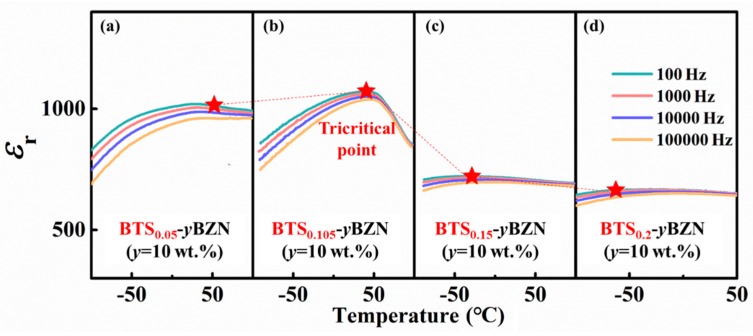
Temperature-dependence of dielectric permittivity for BTS*_x_*-*y*BZN (*y* = 10 wt %) (**a**) *x* = 0.05; (**b**) *x* = 0.105; (**c**) *x* = 0.15; (**d**) *x* = 0.2 at different testing frequencies (*f* = 10^2^, 10^3^, 10^4^, 10^5^ Hz). *x* = 0.105 shows the maximum peak value, indicating the tricritical behavior for this composition.

In order to discuss the role of tricritical transition on the dielectric response for BZN-doped BaTiO_3_–based ceramics, the thermal spectrum of dielectric permittivity for the specimens has been measured with the composition across the tricritical composition. The tricititical transition [27,28,29,30], which is the crossover point between the first-order and second-order phase transition, manifests itself as the enhancement of the dielectric permittivity maximum at Curie temperature when the composition or stress is approaching to it [31]. Originally, the tricritical point has been reported in BaTiO_3_ and PbTiO_3_ systems under hydrostatic pressure [32,33]. Later, such a phenomenon has been reported in the composition–temperature phase diagram of the Pb(Zr_1−*x*_Ti*_x_*)O_3_ (PZT) system [34]. A recent investigation on the high-performance BaTiO_3_-based ceramics suggests that a large dielectric response with the maximum permittivity *ε_r_* > 20000 appeared at the Curie temperature of tricritical composition [7,8]. Further studies reveal that higher dielectric permittivities (*ε_r_* = 45000 ~ 54000) can be achieved in the tricritical point of BTS and Ba(Ti_1−*x*_Hf*_x_*)O_3_ (BTH) ceramics [9,10,35]. The temperature-dependence of dielectric permittivity for BTS*x*-10BZN is shown in Figure 4. It can be seen that when the composition approaches the tricritical point at *x* = 0.105, the dielectric permittivity reaches to the maximum peak value of 1264. Therefore, the results verify that the BZN addition has not changed the tricritical point composition of the BTS system. It should be noted that the pure BZN system showed a relatively low dielectric permittivity of around 100~160 for thick film, as well as ceramics, as reported by Wang’s group [23,24], and therefore the addition of tricritical ferroelectric material can largely enhance the dielectric permittivity for the material system. In addition, the dielectric loss tan*δ* for all of our specimens was less than 5% in a temperature range of −50 °C to 100 °C, and a frequency range of 10^2^−10^5^ Hz.

### 3.5. Electric Breakdown

**Figure 5:**
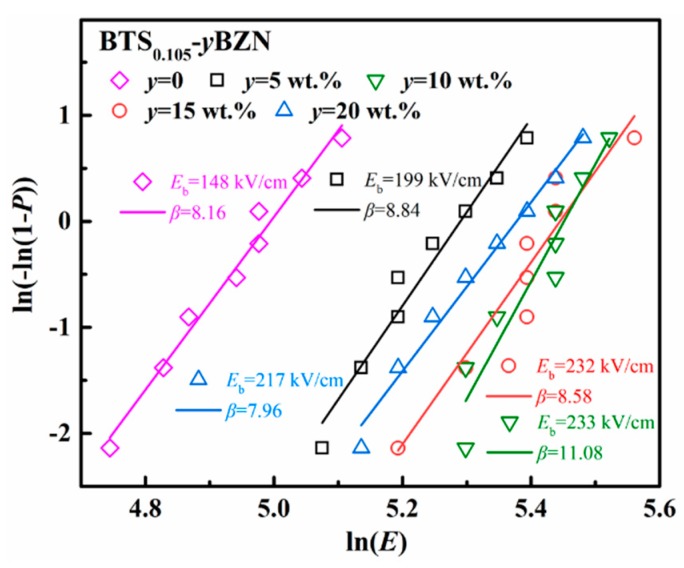
Weibull analysis for the BTS_0.105−_*y*BZN ceramics (*y* = 5 wt %, 10 wt %, 15 wt %, 20 wt %) describing the electric strength for the materials.

In order to evaluate the role of BZN on tuning the electric breakdown for the barium titanate ceramic system, the AC breakdown strength test was performed for BTS_0.105−_*y*BZN ceramics with different BZN contents. Wu et al. reported that the addition of BZN could largely enhance the dielectric strength of pure BaTiO_3_ ceramics [22]. This study suggests that such a method is also effective in the case of tricritical ferroelectrics. The breakdown behavior for tricritical ferroelectrics has been analyzed by using a two-parameter Weibull distribution relationship: *P =* 1 − exp(−*E*/*E*_b_), where *E* is the tested breakdown strength, and *P* is the possibility of electric failure. *E*_b_ and *β* are the scale and shape parameters, which describe the statistical dielectric strength and the dispersion of the data, respectively. The tested results are shown in Figure 5. The BTS_0.105_ ceramic without the addition of BZN showed a relatively low breakdown strength of *E*_b_=148 kV/cm (with the shape parameter *β* = 8.16). When adding 5 wt % content of BZN, the breakdown strength of the specimen rose to *E*_b_ = 199 kV/cm (with the shape parameter *β* = 8.84). On further increasing the content of BZN to 10 wt %, the breakdown strength will be enhanced to a maximum value of *E*_b_ = 233 kV/cm. It is obvious that the addition of BZN can effectively enhance the breakdown strength by 57% for tricritical ferroelectric BTS_0.105_ materials. It is highly possible that the addition of BZN, exhibiting an intermediate dielectric permittivity (*ε_r_* = 30 ~ 160), is able to smooth the electric field distribution around the high-permittivity tricritical-ferroelectric grains [23]. Further increasing the BZN content to 15 wt % or 20 wt % will lead to a slight decrease of dielectric strength, with *E*_b_ = 232 kV/cm and 199 kV/cm, respectively. The possible reason may lie in the slight drop of density with the addition of BZN. It can thus be concluded that BZN additives can effectively enhance the breakdown strength for tricritical ferroelectrics.

### 3.6. Energy Density and Energy Efficiency

**Figure 6:**
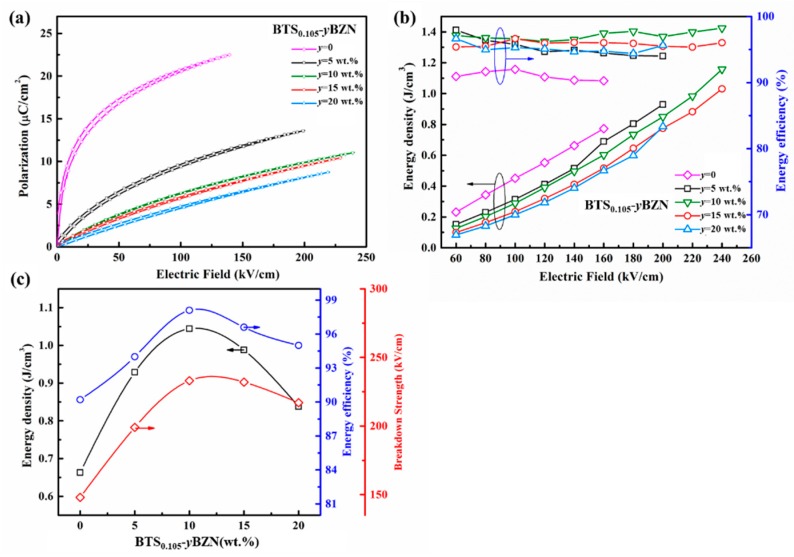
The energy storage properties for BTS_0.105−_*y*BZN (*y* = 5 wt %, 10 wt %, 15 wt %, 20 wt %) ceramics. (**a**) *P-E* hysteresis loops; (**b**) electric field strength-dependence of energy density and efficiency; (**c**) The change of breakdown strength, energy density, and efficiency with BZN content.

The energy storage performance for the BTS_0.105−_*y*BZN ceramics can be evaluated by measuring the polarization-electric field curves (known as *P-E* hysteresis loops) for the specimens. As shown in Figure 6a, the *P-E* loop for BTS_0.105_ without BZN doping shows a large maximum polarization of *P*_max_ ≈ 23 μC/cm^2^, but the specimen can withstand a relatively low electric field strength, and the polarization saturates at a comparably low field region. With an increase in the BZN content, although the maximum polarization decreases to less than 10 μC/cm^2^, the maximum electric field increases.

In order to further qualify the energy performance for the BTS-BZN system, the energy density for the BTS_0.105_-*y*BZN specimens has been calculated by the integration of the *P-E* curve with respect to the electric field from *P*_max_ to *P*_r_, which is an indication of energy that can be release from the material with the removal of the electric field. As shown in Figure 6b, all of the compositions show a similar tendency for the energy density to increase with an increase of the electric field, and the maximum value of energy density for this material system appears at a composition of *x* = 0.105, *y* = 10 wt %, and the energy density value can reach to *U*_e_ = 1.15 J/cm^3^. Such a result is slightly higher than the previously-reported BT-BZN system, with a maximum discharging energy density of 0.8 J/cm^3^, as reported by Wu L.W. et. al [22]. Although such a level of energy density is lower than the polymer/BaTiO_3_ composite materials (with an energy density of around 7 J/cm^3^ [21,36]), it can still be considered as one of the more promising ceramic capacitor materials, since the value is superior to the value (*U*_e_ < 0.035 J/cm^3^) that was first reported in BTS_0.105_ ceramics [9], which indicates the possibility for tricritical ferroelectrics as a promising material system for future energy storage devices.

The composition dependence of energy density, breakdown strength, and efficiency has been shown in Figure 6c. It can be seen that the breakdown strength firstly increases and then decreases with the increase of BZN content, and the optimal breakdown strength is found at a BZN content of 10 wt %. Concerning the composition-dependence of energy storage performance, it shows a similar tendency to breakdown strength, and the maximum energy density appears at a BZN content of 10 wt %. The consistency between the *U*_e_ and *E*_b_ suggests that the energy density of tricritical ferroelectrics is effectively improved through enhancing the breakdown strength via the addition of BZN. Furthermore, such a BTS_0.105−_*y*BZN material system shows a high efficiency of up to 99%, which confirms the advantage for the possible application of tricritical ferroelectrics.

## 4. Conclusions

In conclusion, an efficient method has been applied to achieve improved energy density and ultrahigh energy efficiency for ferroelectric materials by doping BZN in tricritical ceramics. The doped BZN can obviously lower the sintering temperature of BTS ceramics. Furthermore, the highest electrical breakdown strength appeared in BTS_0.105−_*y*BZN (*y* = 10 wt %), with a value of *E*_b_ = 233 kV/cm. On this basis, the relative enhanced energy density reached *U*_e_ = 1.15 J/cm^3^, which may be ascribed to the formation of a merged structure. On the other hand, the relative energy efficiency is more than 99 %, which may be related to the tricritical phenomenon. Hence, doping BZN in tricritical ceramics is a significant approach for improving the energy performance for materials.
